# Correlations of Life Form, Pollination Mode and Sexual System in Aquatic Angiosperms

**DOI:** 10.1371/journal.pone.0115653

**Published:** 2014-12-19

**Authors:** Zhi-Yuan Du, Qing-Feng Wang

**Affiliations:** Key Laboratory of Aquatic Botany and Watershed Ecology, Wuhan Botanical Garden, Chinese Academy of Sciences, Wuhan, China; The National Orchid Conservation Center of China; The Orchid Conservation & Research Center of Shenzhen, China

## Abstract

Aquatic plants are phylogenetically well dispersed across the angiosperms. Reproductive and other life-history traits of aquatic angiosperms are closely associated with specific growth forms. Hydrophilous pollination exhibits notable examples of convergent evolution in angiosperm reproductive structures, and hydrophiles exhibit great diversity in sexual system. In this study, we reconstructed ancestral characters of aquatic lineages based on the phylogeny of aquatic angiosperms. Our aim is to find the correlations of life form, pollination mode and sexual system in aquatic angiosperms. Hydrophily is the adaptive evolution of completely submersed angiosperms to aquatic habitats. Hydroautogamy and maleflower-ephydrophily are the transitional stages from anemophily and entomophily to hydrophily. True hydrophily occurs in 18 submersed angiosperm genera, which is associated with an unusually high incidence of unisexual flowers. All marine angiosperms are submersed, hydrophilous species. This study would help us understand the evolution of hydrophilous pollination and its correlations with life form and sexual system.

## Introduction

Aquatic plants are plants that have adapted to living in aquatic environments (saltwater or freshwater). These plants require special adaptations for living submersed in water, or at the water surface [1], [2]. Vascular aquatic plants are interpreted as all Pteridophyta and Spermatophyta whose photosynthetically active parts are submersed in water or float on the surface of water [1]. Aquatic plants are phylogenetically well dispersed across the angiosperms, with at least 50 origins, although the fraction of species probably comprises less than two percent of the angiosperm species [1], [3]. Although aquatic plants are typically discussed as a unified biological group, the ways that species have evolved to live in the aquatic environment are as diverse as the different evolutionary lineages that became aquatic [4], [5].

Aquatic plants have evolved from often very different genetic and ecological backgrounds. Also, they have evolved at different times; some old ones are aquatic at the level of order or family, while others, more recent, are isolated species in otherwise terrestrial genera [6]. Some shared traits are likely independently evolved in multiple lineages [7]. For example, a transition to the aquatic habit in both water lilies and *Ceratophyllum* may have led to convergent evolution of traits associated with the aquatic lifestyle, such as absence of a vascular cambium, highly dissected leaves, and high photosynthetic rates [7]. Reproductive and other life-history traits of aquatic angiosperms are closely associated with specific growth forms: emerged from the water, free-floating, floating-leaved, or submersed [4]. These categories represent different degrees of adaptation to aquatic life and are widely convergent among aquatic angiosperms [4].

Hydrophilous pollination exhibits notable examples of convergent evolution in angiosperm reproductive structures. Two general classes of hydrophilous pollination (hydrophily) occur in angiosperms: ephydrophily and hyphydrophily [8]. Ephydrophily is pollination at the water surface, e.g., *Vallisneria* (Hydrocharitaceae). Hyphydrophily occurs among flowers that are completely submersed, e.g., *Zostera* (Zosteraceae). Hydrophily represents a remarkable evolutionary departure from the pollination systems of terrestrial plants [9]. In the strictest sense (release and capture of wet, water-borne pollen), this abiotic pollination system entails structural and biochemical modifications of aerial pollination systems and the complete abandonment of aerial flowers [9].

True hydrophily occurs in 18 submersed angiosperm genera, which exhibit great diversity in sexual system [3]. Two genera include hermaphroditic species, 7 have monoecious species, and 11 have dioecious species. The prevalence of dicliny in hydrophiles has led to assumptions of outcrossing and high levels of genetic variability in these plants [10]. Although most aquatic plants can reproduce by flowering and setting seed, many also have extensive asexual reproduction by means of rhizomes, turions, and fragments [10]. The predominant role of asexual reproduction and clonal growth in many hydrophile populations may restrict the degree of outcrossing [10]. This dilemma between dicliny and outcrossing may be resolved by taking into account the early evolution of hydrophilous plants [10].

In this study, we reconstructed ancestral characters of aquatic lineages based on the phylogeny of aquatic angiosperms. The purpose of this article is to look for the correlations of life form, pollination mode and sexual system in aquatic angiosperms. Specifically, we ask: what is the relationship between hydrophily and aquatic life forms, and what is the relationship between hydropily and sexual systems? As angiosperms diversified and flourished in freshwater habitats, some species ultimately colonized marine environments and became seagrass. In this study, we also want to explore its relationship among above characters.

## Materials and Methods

In a study of the origin times and areas of different aquatic plant lineages, we used two chloroplast gene sequences (*rbc*L and *mat*K) of 305 aquatic species to construct a phylogenetic tree of aquatic plants (in manuscript). We sampled 100 species representing 67 genera of aquatic plants from China. We also obtained the sequences of 205 species representing other 72 aquatic genera from the Genbank. These 139 genera (in 43 families) represent the majority of the families of aquatic plants that obligately live in water. Amphibious plants are distinct from aquatic species that live constantly in water, and thus most amphibious species were not included. Phylogenetic analyses were conducted using maximum likelihood methods and Bayesian methods. In that study, we estimated the divergence times and constructed the ancestral areas to get the origin times and areas of different aquatic plant lineages.

In this study, we derived a summary tree at the generic level from the phylogenetic relationships of the aquatic species. Our analysis includes all but one of the hydrophilous genera (*Althenia*). The topology of aquatic genera was applied to reconstruct ancestral character states. We explored the evolutionary transitions of life forms, pollination modes and sexual systems in aquatic angiosperms using Mesquite v.2.74 [11]. These analyses permitted estimates of numbers, directionality, and timing of transitions among each character states.

In this study, aquatic life forms had five states: helophyte, emergent (the roots and base of the plant are submersed, but some photosynthetic parts and sexually reproductive parts are emergent), floating -leaved (the roots and base of the plant are submersed, but some photosynthetic parts are floating on the water surface and sexually reproductive parts are emergent), submersed (the roots and photosynthetic parts of the plant are submersed, and sexually reproductive parts are submersed or emergent) and free-floating (without roots or with roots hanging in the water column) [12]. The pollination modes of aquatic plants had six states: entomophily, anemophily, ephydrophily, hyphydrophily, maleflower-ephydrophily and hydroautogamy. The sexual systems of aquatic plants had five states: hermaphroditism, monoecy, dioecy, andromonoecy and gynodioecy. Character states of each genus were scored based on the study of Cook [1] and field observations.

## Results

### The reconstruction of life forms

**Figure 1:**
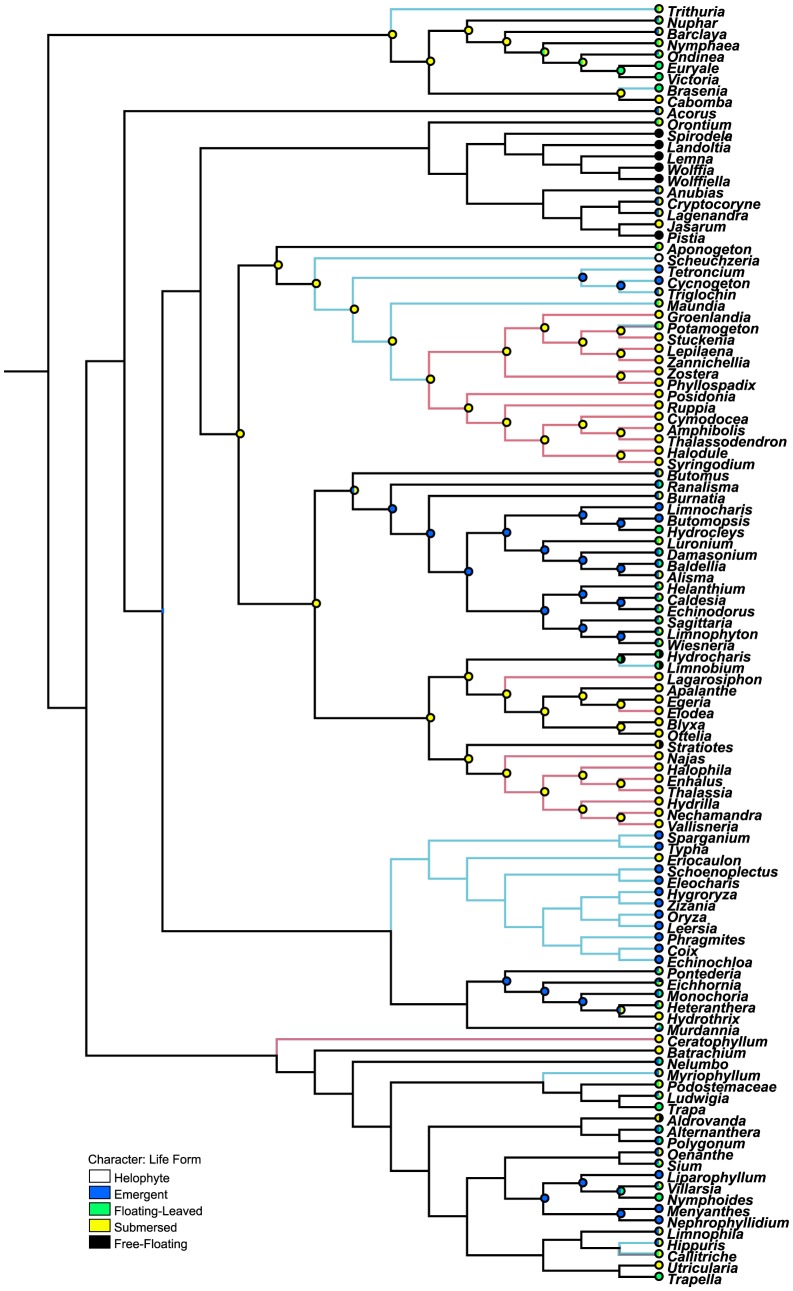
Life form reconstruction of aquatic families. Bars at nodes represent the ancestrial states. Blue lines represent anemophilous pollination modes. Red lines represent intermediate and true hydrophilous pollination modes. *Potamogeton* and *Callitriche* have both colors of lines.

The aquatic angiosperms split into three lineages, and the four aquatic orders are at the basal node of each lineage (Fig. 1). The growth form of Acorales is submersed-emergent, and the growth form of Ceratophyllales is submersed. Character state analysis of the aquatic growth form showed that the ancestral habit of Nymphaeales was likely submersed, which gave rise to the floating-leaved forms. The reconstruction analysis also indicates an ancestral state of submersed life-form in core Alismatales. The result suggests multiple origins of floating-leaved aquatics in core Alismatales involving Alismataceae, Hydrocharitaceae, Aponogetonaceae, Juncaginaceae and Potamogetonaceae. The reconstruction indicates three independent origins of the emergent life-form in core Alismatales (Butomaceae, Alismataceae and Juncaginaceae). The rare free-floating life form in the core Alismatales likely evolved from the submersed life form (in *Stratiotes*) or the floating leaved life-form (in *Limnobium* and *Hydrocharis*). Arace have all the four aquatic life forms.

There are five aquatic families in terrestrial orders (Fig. 1). The growth forms of Typhaceae and Nelumbonaceae are emergent. The emergent-submersed life form was suggested as the progenitorial state of Pontederiaceae. The progenitorial state of Menyanthaceae is emergent, which gave rise to floating-leaved life form. Podostemaceae has submersed life form. These aquatic families may transit from terrestrial plants and gradually adapt to the aquatic habitats. Most aquatic species in poales are emergent, and all the four growth forms appear in other eudicot genera.

### The reconstruction of pollination modes

**Figure 2:**
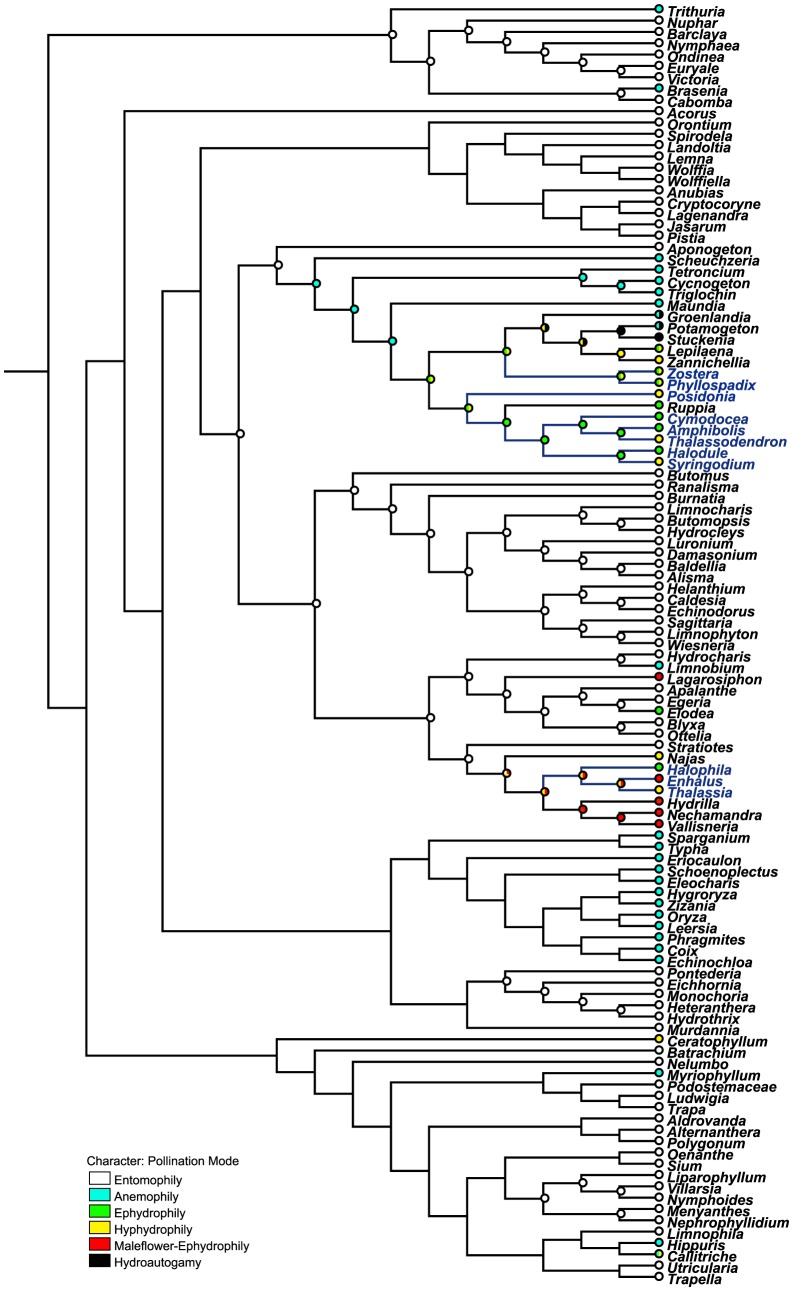
Pollination mode reconstruction of aquatic families. Bars at nodes represent the ancestrial states. Deep blue words and lines represent marine genera.

Wind pollination in aquatic plants has originated several times from diverse lineages. There are eleven anemophilous families in aquatic plants (Fig. 2): Hydatellaceae, Scheuchzeriaceae, Potamogetonaceae, Juncaginaceae, Maundiacese, Eriocaulaceae, Poaceae, Cyperaceae, Typhaceae, Haloragaceae and Plantaginaceae. The broad systematic distribution of anemophily indicates a polyphyletic history.

Hydrophily is polyphyletic with at least eight separate origins (families), including six separate origins of hydrophily in Alismatales, with only a few species found in Ceratophyllaceae and Callitrichaceae (Fig. 2). In Alismatales, six families containing hydrophilous species are Hydrocharitaceae, Posidoniaceae, Ruppiaceae, Cymodoceaceae, Potamogetonaceae and Zosteraceae.

Only 18 aquatic genera have acquired true hydrophily, and the other seven genera with maleflower-ephydrophily or hydroautogamy are at the transitional states to true hydrophily. Maleflower-ephydrophily had two separate origins in two clades of the entomophilous family Hydrocharitaceae. Hydroautogamy evolved in the anemophilous family Potamogetonaceae. The rest aquatic families are entomophily, such as Nymphaeaceae, Acoraceae, Araceae, Butomaceae, Alismataceae, Aponogetonaceae and Pontederiaceae. Most of the eudicot genera are also insect-pollinated.

### The reconstruction of sexual system

Nymphaeaceae, Cabombaceae, Acoraceae, Scheuchzeriaceae, Maundiacese, Posidoniaceae, Ruppiaceae, Butomaceae, Pontederiaceae and most of the eudicot genera are hermaphroditic. Aponogetonaceae, Zosteraceae, Cymodoceaceae, Typhaceae, and Ceratophyllaceae have unisexual flowers. Most of the aquatic genera in Arace are monoecious. The rest families have both bisexual and unisexual flowers, such as Juncaginaceae, Potamogetonaceae, Alismataceae, Hydrocharitaceae, Poaceae and Menyanthaceae. Hermaphroditism was the ancestral state of these families except Hydrocharitaceae. Sexual system reconstruction indicates that dioecy was the ancestral state in Hydrocharitaceae, and monoecy and hermaphroditism were derived from dioecy.

**Figure 3:**
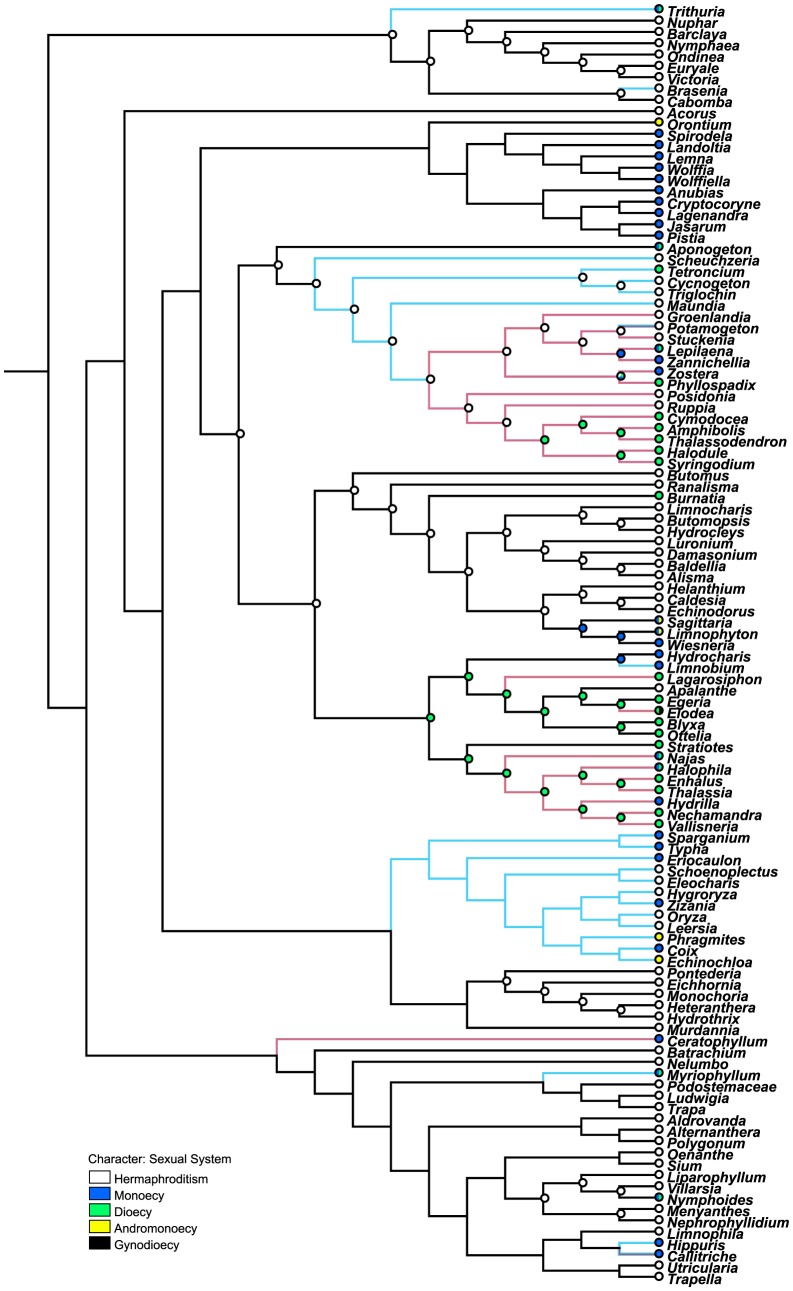
Sexual system reconstruction of aquatic families. Bars at nodes represent the ancestrial states. Blue lines represent anemophilous pollination modes. Red lines represent intermediate and true hydrophilous pollination modes. *Potamogeton* and *Callitriche* have both colors of lines.

Hydrophiles exhibits great diversity in sexual system (Fig. 3). An overwhelming consistency is the diclinous sexual condition (monoecy or dioecy) in true hydrophilous angiosperms, with hermaphroditic flowers occurring in only two genera, *Posidonia* (Posidoniaceae) and *Ruppia* (Ruppiaceae). *Ceratophyllum* (Ceratophyllace), *Callitriche* (Callitrichaceae), *Zannichellia* (Potamogetonaceae) and *Zostera* (Zosteraceae) are monoecious. All the five genera of Cymodoceaceae, *Phyllospadix* (Zosteraceae), *Elodea* and *Thalassia* (Hydrocharitaceae) are dioecious. The rest three genera *Lepilaena* (Potamogetonaceae), *Najas* and *Halophila* (Hydrocharitaceae) have both monoecious and dioecious species.

## Discussion

### Life form evolution of aquatic angiosperms

The overall phylogeny of aquatic angiosperms was congruent with other angiosperm phylogenetic studies. The aquatic angiosperms split into three lineages, and the aquatic orders are at the basal node of each lineage. Nymphaeales diverged from the basal node of the extant aquatic angiosperms. Aquatic monocots and eudicots are well supported as monophyletic. Acorales and Alismatales are successive sister groups of the remaining monocots. *Ceratophyllum* is placed sister to eudicots. The submersed life form was suggested as the progenitorial state of these aquatic orders, which gave rise to floating-leaved, free-floating and emergent life forms with multiple origins of each life form. Their ancestral life form is in accordance with their aquatic origins.

Most of the aquatic families in terrestrial orders evolved from emergent life form. Their ancestral life form is also consistent with their terrestrial origins. The aquatic genera and species in terrestrial families provide evidence of evolution from emergent to floating-leaved or submersed life forms, which indicates an evolutionary transition from terrestrial ancestors through amphibious state to fully aquatic habit [13].

### Evolution of hydrophilous pollination

The first angiosperms were insect-pollinated, and anemophily is a derived state [14]. Insect pollination was suggested as the progenitorial state of Nymphaeales and Alismatales, which gave rise to wind pollination with multiple origins. In only two genera, *Brasenia* (Cabombaceae) and *Limnobium* (Hydrocharitaceae), is it likely that the evolution from entomophily to anemophily has taken place in the aquatic environment [14].

Hydrophily is unique to submersed aquatic angiosperms and characterizes only 26 of approximately 102 genera of obligate aquatic angiosperms [15]. Two classes of true hydrophily are recognized: ephydrophily (pollination at the water surface) and hyphydrophily (pollination below the water surface). However, it is evident that ephydrophily embraces two rather dissimilar subtypes [16]. In true ephydrophily (wet-ephydrophily), the reproductive structures are wet at anthesis but in close association with the water surface, e.g., the pollen floats just below the water surface but not upon it [16]. In maleflower-ephydrophily (dry-ephydrophily), the flowers undergo anthesis above the water surface and the reproductive structures (stigma and pollen) remain dry. Convergent hydrophilous pollination has evolved from both biotic and abiotic pollination systems, and two models for the evolution of hydrophily were proposed: the surface-intermediate model and the selfing-intermediate model [4].

#### The surface-intermediate model of hydrophily from entomophily

Pollination mode in Hydrocharitaceae ranges from entomophily, anemophily, maleflower-ephydrophily, ephydrophily to hyphydrophily. Maleflower-ephydrophily is an intermediate stage between insect pollination and true water pollination in aquatic plants. This intermediate condition consists of flowers that retain entomophilous features such as showy petals but are pollinated by detached, floating male flowers that directly contact stigmas of female flowers with their anthers. The underwater male inflorescences rupture at maturity, and the unopened male flowers float to the water surface. The floating female flowers are anchored by a long peduncle. The stigma and inside of the sepals are unwettable and create a slight depression in the water surface into which the male flowers tumble. After fertilization, the peduncle draws the female flower under water where the fruit develops. There are four dioecious Hydrocharitaceae genera with maleflower-ephydrophily: *Lagarosiphon*, *Enhalus*, *Vallisneria* and *Nechamandra*.

Unlike the above four genera, the floating male flowers of *Hydrilla* (Hydrocharitaceae) aerially discharge pollen to the stigmas of female flowers [8]. However, *Hydrilla* is not strictly wind pollinated, because heavy pollen grains are actively propelled from the male flowers to the females and wind plays an unimportant role [14]. For effective pollination, the pollen of *Hydrilla* should not get into the airstream, as in other anemophilous plants, but must drop almost vertically to reach the stigmas. This unique mechanism relies on water, and therefore probably evolved in water [14].

#### The selfing-intermediate model of hydrophily from anemophily

Hydroautogamy is theorized as an intermediate stage between wind and water pollination in aquatic plants [3]. Hydroautogamy describes self-pollination wherein pollen is transported upon the surface of an air bubble from the anther to the stigma of the same flower [15], [16]. During anthesis, bubbles are produced as gas is released from the dehiscing anthers. Pollen travels from the anther onto the outer surface of the bubble. The bubble increases in size until it extends from the anther to the stigma. Pollen is then deposited onto the stigma from the bubble surface and self-pollination occurs. The bubble continues to enlarge until it breaks free from the flower and rises to the surface [16]. Self-pollination occupies a key intermediate position that allows hydrophilous features to accumulate while maintaining seed production [4].

Hydroautogamy have been reported in three hermaphroditic genera of Potamogetonaceae [4], [17], [18]. In these genera, another uncommon pollination phenomenon was reported. Their pollen would fall on the water surface after release and float in clumps. Occasionally some emergent inflorescences may sink below the water surface by water currents, and their stigmas would come in close contact with the floating clumps and pick up pollen grains directly from the water surface. Whether this is true ephydrophily is still under debate [3], but it is no doubt an important transitional stage for true ephydrophily.

Analogous systems of hydroautogamy that involve self-pollination between unisexual flowers on a plant (geitonogamy) have been described in the dicot genus *Callitriche* [2]. This kind of internal geitonogamy involves pollen tube growth from within anthers of staminate flowers, through vegetative tissues, into the base of pistillate flowers [2]. It is thought to be the transitional stage between anemophilous and hydrophilous species in *Callitriche*.

#### The role of the two intermediate pollination modes

Maleflower-ephydrophily and hydroautogamy occupied key intermediate stages between aerial systems and true hydrophily. The system of floating flower reproduction in the Hydrocharitaceae could predictably lead to repeated contact of pollen and stigma with water and may select for characters to resist or accommodate wettability [4]. In hydroautogamy, flowers open while submersed. Under this circumstance, pollen and stigma are exposed to water during anthesis. Such intermediates would provide opportunities for the operation of selective pressures leading to the gradual accumulation of two major characters that distinguish true hydrophily from aerial systems: wettable pollen and wettable stigma [16].

When the intermediate and true hydrophilous pollination modes are considered together, there are two hydrophilous clades in Alismatales (Figs.1–3). One is comprised of Potamogetonacea, Zosteraceae, Posidoniaceae, Ruppiaceae and Cymodoceaceae. Another clade is Hydrocharitaceae. These two hydrophilous clades are in accordance with the two gradual models, which indicate two independent origins of hydrophily in Alismatales from anemophily and entomophily respectively.

### Sexual system evolution of aquatic angiosperms

Dioecy in angiosperms is phylogenetically widespread, with probably more than 100 distinct origins, but is also relatively rare, accounting for only 6% of all species [19], [20]. Dioecy is normally considered as a derived state within angiosperms [21]. The evolution of dioecy from cosexuality is considered to occur via gynodioecy or monoecy [22]. Although it is less common than the transition from hermaphroditism to dioecy, cosexuality has been inferred to be a potentially derived state in several angiosperm lineages [22]. Our results indicate that monoecy and hermaphroditism were derived from dioecy in Hydrocharitaceae, which provides evidence of reverse evolution from dioecy to hermaphroditism.

Unisexual flowers characterize most of the cases of anemophily and hydrophily. Anemophily is associated with either spatial or temporal separation of male and female reproductive structures, such as monoecy, dioecy and dichogamy (phenological separation between pollen release and pollen reception), which may encourage outcrossing [20]. As in the case of anemophily, most hydrophilous flowers are monoecious, dioecious, or dichogamous [3], which would facilitate outcrossing by effectively separating the pollen and stigmas of the same flower or inflorescence [23]. However, there is also a high degree of clonality associated with hydrophilous plants, which may restrict outcrossing [10]. This appears to be due to the systematic affinity of the taxon [23]. Specifically, clonal growth is quite common within the monocots [24].

### Correlations of unisexuality and hydrophily

More than 90% of hydrophilous species possess unisexual flowers [10]. There were two different views about the correlations of unisexuality and hydrophily. Philbrick suggested that unisexuality was acquired not before, but after the initial submergence of a bisexual flower and initial selection toward hyphydrophily was on a bisexual flower [16]. Les hypothesized that unisexuality in at least some hydrophiles may simply reflect the sexual condition of their progenitors rather than represent an adaptation linked to water pollination [10], that is, unisexuality occurs before hydrophily. There are evidences for both views, which are related with the two gradual models of hydrophily.

In the selfing-intermediate model of hydrophily from anemophily, the wettablity of both pollen and stigma may acquire after the emergent bisexual flowers are repeatedly submersed by water currents. Because the exclusively hydrophilous clade of Cymodoceaceae complex (Posidoniaceae, Ruppiaceae, and Cymodoceaceae) is hermaphroditic primitively, it appears that bisexual hydrophiles have preceded unisexual hydrophiles evolutionarily. In addition, true hydrophily and unisexuality derived from hydroautogamy and hermaphroditism in Potamogetonaceae. These evidences conform to Philbrick's hypothesis that initial selection toward hydrophily was on a bisexual flower [16].

In the surface-intermediate model of hydrophily from entomophily, the wettablity of both pollen and stigma may acquire after the floating unisexual flowers repeatedly submerging in the water. Simultaneous and independent acquisition of hydrophilous features in both staminate and pistillate flowers may merge within unisexual species after fertilization. Unisexuality is a primitive rather than derived state in the Hydrocharitaceae, and all the intermediate and true hydrophilous genera in this hydrophilous clade are unisexual. Therefore, the unisexual conditions of all hydrophilous species in this family are, as Les hypothesized, a consequence of a pre-existing state in their non-hydrophilous progenitors [10]. The case in Hydrocharitaceae supports Les's hypothesis [10].

In summary, hydrophily may evolve in both bisexual and unisexual flowers, and unisexuality can be both ancestral condition and derived state in hydrophiles. These disparate results emphasize the complexity of adaptive evolution in hydrophiles, which has occurred along convergent pathways [3].

### Correlations of life form and pollination mode in marine angiosperms

Seagrasses are a functional group of about 50 species in 11 genera and five families [3]. There are essentially no marine bryophytes, pteridophytes or gymnosperms [1]. The reconstruction results indicate there to be three independent origins of seagrasses, and the important evolutionary affinity between water pollination and the ability of angiosperms to colonize marine habitats (Fig. 2). All marine angiosperms are submersed, hydrophilous species. This is because subtidal seagrass populations exist at depths where only hydrophily is possible [25]. Seagrasses possess a number of morphological features that appear to be associated with hydrophily. Specifically, their pollen is unique and has evolved convergently to filamentous shapes or functionally-filamentous [26]. From the subset of submersed, hydrophilous angiosperms, only 11 genera have colonized marine habitats. Among them, 10genera are true hydrophilous, and only one marine genus *Enhalus* is maleflower-ephydrophily, a transitional state of true hydrophily.

All marine angiosperms are exclusively from Alismatales. Arber recognized that the limited diversity of marine flora was the result of their need to evolve four special faculties [27]: (1) toleration towards a saline medium; (2) the power of vegetating while wholly submersed; (3) the knack of developing a sufficiency of anchoring roots to withstand the action of waves and tides; (4) the capacity for hydrophilous pollination. Seagrasses appear to have evolved a unique set of physiological abilities related to salt tolerance that are different from those of their freshwater relatives [25]. Both water pollination and salt tolerance represent difficult evolutionary transitions for angiosperms to colonize marine habitats [3]. Submersed, salt-tolerant, and hydrophilous species occur only in three angiosperm groups (Alismatales, Ceratophyllaceae and Callitrichaceae) [3]. Arber attributed the lack of marine species in the other two groups to the lack of rhizomes in Callitrichaceae and absence of roots in Ceratophyllaceae [27]. Most species in Alismatales are rhizomatous perennials with effective anchorage systems, which make Alismatales the only group that have colonized marine habitat.

**Figure 4:**
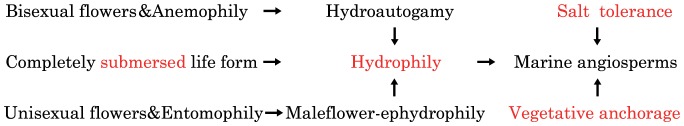
The correlations of life form, sexual system, pollination mode and marine angiosperms. Red words represent the four requisites of marine angiosperms.

The correlations of life form, pollination mode, sexual system and marine angiosperms are summarized in Fig. 4.

## Conclusions

The pollination modes of aquatic angiosperms are closely associated with specific growth forms. Entomophily and anemophily occur in all the life forms with sexually reproductive parts held above the water or at the surface. Hydrophily is unique to obligate submersed aquatic angiosperms with sexually reproductive parts completely submersed below the water surface. Hydrophily is the adaptive evolution of completely submersed angiosperms to aquatic habitats. Hydroautogamy and maleflower-ephydrophily are the transitional stages of hydrophily which evolved from anemophily and entomophily respectively. True hydrophily occurs in 18 submersed angiosperm genera, which is associated with an unusually high incidence of unisexual flowers. The three hydroautogamous genera in Potamogetonaceae are bisexual, and the five maleflower-ephydrophilous genera in Hydrocharitaceae are dioecious. All marine angiosperms are submersed, hydrophilous species and are known only from Alismatales.
